# The Effects of Either a Mirror, Internal or External Focus Instructions on Single and Multi-Joint Tasks

**DOI:** 10.1371/journal.pone.0166799

**Published:** 2016-11-29

**Authors:** Israel Halperin, Steven Hughes, Derek Panchuk, Chris Abbiss, Dale W. Chapman

**Affiliations:** 1 Physiology Discipline, Australian Institute of Sport, Canberra, Australian Capital Territory, Australia; 2 Centre for Exercise and Sport Science Research, School of Medical & Health Sciences, Edith Cowan University, Joondalup, Western Australia, Australia; 3 Institute of Sport, Exercise & Active Living, College of Sport & Exercise Science, Victoria University, Footscray, Victoria, Australia; 4 Skill Acquisition Discipline, Australian Institute of Sport, Canberra, Australian Capital Territory, Australia; Victoria University, AUSTRALIA

## Abstract

Training in front of mirrors is common, yet little is known about how the use of mirrors effects muscle force production. Accordingly, we investigated how performing in front of a mirror influences performance in single and multi-joint tasks, and compared the mirror condition to the established performance effects of internal focus (IF) and external focus (EF) instructions in a two part experiment. In the single-joint experiment 28 resistance-trained participants (14 males and 14 females) completed two elbow flexion maximal voluntary isometric contractions under four conditions: mirror, IF, EF and neutral instructions. During these trials, surface EMG activity of the biceps and triceps were recorded. In the multi-joint experiment the same participants performed counter-movement jumps on a force plate under the same four conditions. Single-joint experiment: EF led to greater normalized force production compared to all conditions (*P*≤0.02, effect-size range [ES] = 0.46–1.31). No differences were observed between neutral and mirror conditions (*P* = 0.15, ES = 0.15), but both were greater than IF (*P*<0.01, ES = 0.79–1.84). Surface EMG activity was comparable across conditions (*P*≥0.1, ES = 0.10–0.21). Multi-joint experiment: Despite no statistical difference (*P* = 0.10), a moderate effect size was observed for jump height whereby EF was greater than IF (ES = 0.51). No differences were observed between neutral and mirror conditions (ES = 0.01), but both were greater than IF (ES = 0.20–22). The mirror condition led to superior performance compared to IF, inferior performance compared to EF, and was equal to a neutral condition in both tasks. These results provide novel and practical evidence concerning mirror training during resistance type training.

## Introduction

Over the past two decades a large body of research has investigated the effects of attentional focus conditions on motor learning and performance [1, 2]. Specifically, the effects of instructions that elicit an internal focus (IF) or external focus (EF) of attention on exercise performance have been commonly compared. IF leads individuals to focus on a specific body part or muscle group, whereas EF leads individuals to focus on the intended effects of their movements on the environment. Generally, research has reported that EF enhances motor learning performance, when compared with IF instructions, and compared to neutral instructions, which are deprived of an internal or external point of reference [2]. For example, instructing participants to focus on the movement of their wrist during a basketball shot hinders accuracy, when compared with focusing on the basket [3]. Superior performance with EF is observed with tasks requiring large power output, such as long jump [4], sprint running [5], and in tasks requiring maximal force such as single joint elbow flexion [6], and multi-joint exercises, such as the isometric mid-thigh pull [7]. While preference for instructions/focus conditions has been shown to have a small effect on performance [8, 9], the benefits of EF are consistent across tasks, skill level, and age groups [2].

Physical training is commonly performed in front of mirrors in numerous environments, such as fitness gyms, martial arts and dancing studios. Despite the mirrors apparent popularity, the few studies to investigate the influence of mirrors on motor performance have reported mixed results [10–17]. For example, Bennett and Davids [11] observed that novice and intermediate level powerlifters benefited from performing the squat exercise in front of a mirror when asked to descend to a very precise and optimal depth. In contrast, advanced powerlifts remained unaffected by the mirror when completing the same task. Furthermore, while studies have found a mirror improves static balance performance in both young [15] and old [16] adults, other investigators have not observed differences between mirror and no mirror conditions [18]. In regards to dancing, practicing in front of mirrors enhanced learning and performance of a dance sequence among experienced dancers [12], but hindered dancing performance with untrained participants [13]. These experiments demonstrate the inconsistent findings on the effects of mirror training on motor learning and performance. Furthermore, all of the investigated tasks required movement accuracy, precision and balance, yet many gym goers perform motor tasks that require maximal muscular tension in front of mirrors, such as a barbell squat and biceps curls. However, to the best of our knowledge there are currently no studies examining the effects of mirrors on performance during such tasks.

Conflicting findings also exist as to what emotions and perceptions training in front of a mirror elicits. For example, studies have found that exercising in front of mirrors increase self-efficacy [19], have no effect on self-efficacy [20], lead to a self-conscious negative body imagine [21], and elicit negative feelings [22]. An analysis of interviews with dancers reported that mirrors may be a necessary tool to improve dancing technique [23]. Yet within this study the dancers also stated that mirrors can lead to body objectification due to comparisons of oneself to the image in the mirror. It is interesting to consider that the potential self-conscious response elicited by mirrors is also associated with IF, which is known to hinder motor learning and performance [24]. In summary, the relevant literature concerning how mirrors may affect emotions, perceptions, and feelings during physical training is conflicting. Given that mirrors may influence perceptions and emotions as a result of visual feedback during exercise, it is also of interest to understand how exercise in front of mirrors may affect one’s attentional focus and overall performance. Finally, since females were the participants in the majority of described studies above, it is of interest to compare the effects of mirrors on perceptions as well as on performance between the genders.

It is plausible that looking at a mirror focuses one’s attention to the body part or muscle groups being observed, and elicits IF. Conversely, since the body part being observed in the mirror is external to the self the use of mirrors may elicit EF. Thus we sought to directly investigate this question using a two part study design. Specifically, the goals of these experiments were fourfold: the first was to compare the effects of four sets of instructions: IF, EF, Neutral and Neutral with the addition of a mirror, on maximal voluntary isometric contraction (MVIC) and electromyography (EMG) activity of the elbow flexors, as well as on countermovement jumping performance. The second purpose was to examine if participants’ preference for instructions/focus conditions was matched with their performance outcome, as indicated by previous studies [8, 9]. The third was to understand whether the use of a mirror is perceived as either IF or EF by participants by use of a questionnaire. Finally, since most mirror studies used females as participants, a comparison was made between male and female participants since the use of mirrors has a possible gender effect.

## Methods

### Experimental rationale

The chosen experimental design sought to account for what we perceived to be two substantial confounders of reported literature. The use of a dual experiment design with single and multi-joint tasks were firstly chosen as it was of interest to compare tasks that vary in their degrees of freedom of coordination or central nervous system input. That is, will a single-joint task requiring significantly less degrees of freedom relative to the multi-joint task result in different outcomes. Secondly we were concerned with issues of experimental validity versus ecological validity. In the single-joint experiment participants were only able to observe the contracting muscle group with the mirror, thereby increasing the study’s internal validity and attributing any observed effects to the mirror. This design substantially increases the internal validity as any effects on force production can be attributed to the mirror with a greater degree of certainty. However, this design also has low ecological validity, as it is unlikely that people train and practice in their natural environments while only being able to observe the contracting muscles with a mirror. Thus, in the multi-joint experiment we sought to compensate for the low ecological validly of single-joint experiment, and implement a jumping protocol in which participants were able to observe movement of their whole body within the mirror as they performed the requested task. Hence, the multi-joint experiment design represents training environments to a greater extent which enhances the degree of both external and ecological validity.

### Participants

**Fig 1:**
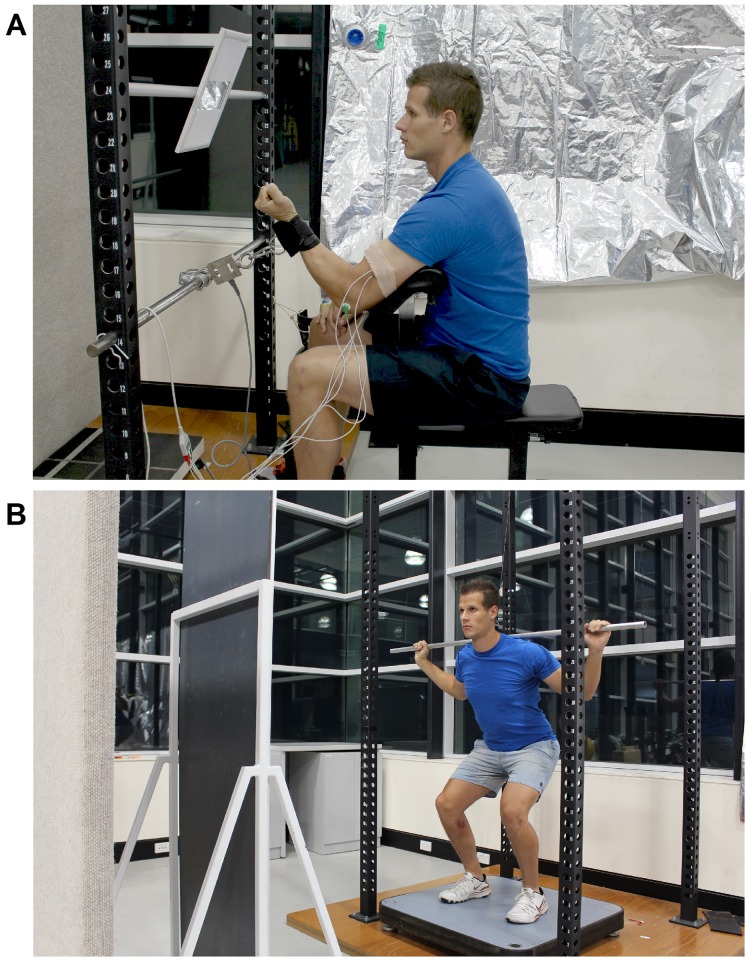
Experimental setup. (A) Single joint experiment. (B) Multi-joint experiment.

Twenty-eight resistance-trained participants volunteered for both experiments (14 males and 14 females, age: 26±5 y, weight: 70±11 kg). All participants had performed resistance training at least twice a week for the past year, and participated in various sporting activities such as Soccer, Rugby and Judo once to three times a week. Participants were provided with a carefully presented verbal description of the study, so as to not compromise the study design. Thereafter, each athlete provided written informed consent. The study was approved by the Australian Institute of Sport Ethics Committee and conformed to the declaration of Helsinki for human research. The individual in Fig 1 has given written informed consent (as outlined in the PLOS consent form) to publish these figures.

Participants attended the laboratory on a single occasion with the maximal voluntary isometric contraction (MVIC) single-joint experiment performed first followed by the countermovement jump (CMJ) multi-joint experiment, after which they completed the questionnaires for both experiments. On arrival each participant was familiarised with the MVIC testing protocol, and thereafter completed the experimental session as described below. During a 5 minute resting period between experiments each participant was familiarised with the CMJ testing protocol, and thereafter completed the CMJ experimental session as described below.

### Single-joint experiment

#### Procedures

All data collection was carried out in a quiet room by the same two investigators, in which the same investigator provided the instructions to all participants. Participants were informed about the importance of maintaining a straight gaze during all trials (other than the mirror condition) with the goal of eliminating possible vision confounders as a result of the instructions. In an attempt to control for gaze, a large mono-tone board was placed in front of participants in both tests to block out potential vision confounders that they may focus on otherwise (Fig 1A).

Prior to initiating the MVIC test, sEMG electrodes were attached to participant’s biceps and triceps brachii muscles. Each participant was then seated on the preacher curl bench (RM, China) with the seat height adjusted so that the elbow joint was at a 90° angle during each isometric contraction and a strap was secured around their wrist which was attached to a force transducer (Fig 1A). Participants then performed a warm up consisting of ten elbow flexion contractions at an intensity equal to ~50% of their perceived maximum (work to rest ratio of 2/2 s) and one 3 s contraction at an intensity equal ~80% of their perceived maximum. Thereafter participants rested for two minutes and then completed two baseline MVICs, lasting 3 s, separated by 30 s of rest. Instructions for the baseline contractions were the same as the Neutral instructions which were “Attempt to produce as much force as you possibly can”. After completion of the second contraction participants were given two minutes of rest. Participants then received one of four instructions in a randomized order prior to completing two MVICs per condition separated by 30 s of rest. Two minutes of rest were provided between each instructional condition.

The instructions provided to each participant are described in Table 1 for each of the investigated conditions. In the Mirror condition a 0.2 x 0.08 m mirror was installed ~0.6 m away from the participants at eye level (Fig 1A). The size of the mirror was constrained so it only allowed participants to see their elbow flexors contracting. To reduce the possibility of participants focusing on the EMG electrodes, a small skin coloured wrap was placed around them. Other than the single instructional sentence no other guidelines, encouragement, verbal or visual feedback were provided.

**Table 1:** Instructions.

Single-joint		
	IF	Attempt to produce as much force as you possibly can while focusing on contracting your arm muscles as hard and as fast as you can
	EF	Attempt to produce as much force as you possibly can while focusing on pulling the strap as hard and as fast as you can
	N	Attempt to produce as much force as you possibly can
	M	Attempt to produce as much force as you possibly can while looking at yourself in the mirror
Multi-joint		
	IF	Attempt to jump as high as you can while focusing on contracting your leg muscles as hard and as fast as you can
	EF	Attempt to jump as high as you can while focusing on pushing of the ground as hard and as fast as you can
	N	Attempt to jump as high as you can
	M	Attempt to jump as high as you can while looking at yourself in the mirror

The instructions provided to each participant in the single and multi-joint experiments for the Internal Focus (IF), External Focus (EF), Neutral (N) and Mirror (M) investigation conditions.

#### Maximum voluntary isometric contraction (MVIC)

Subjects were seated on a preacher curl device with their upper arm supported and elbow flexed at 90°. Secured around the wrist was a padded strap attached by a high-tension wire to a load cell (capacity 200 kg, Sensitivity = 10.2μV/N, Vishay, Australia) to measure elbow flexion forces. All force data were sampled at a rate of 1000 Hz by a personal computer via an 8 channel data acquisition system (PowerLab, ADInstruments, Australia) operated by Labchart software (ADInstruments, Australia) sampling at 1000 Hz, allowing direct measurement of force-time characteristics. Mean force was determined for all contractions. The mean was determined over a 2-s window defined as 0.5 s after the initiating of the contraction and 0.5 before it ended. Due to the expected large inter-subject variability between genders in maximal force production all mean force values were normalized to the baseline condition, and thus reported and analysed as a percentage.

#### Electromyography (EMG)

Surface electromyography (sEMG) recording electrodes (Viasys, USA) were placed approximately 3 cm apart over the proximal, lateral segment of the biceps brachii and over the lateral head of the triceps brachii. Skin preparation included shaving and cleansing of the area with an isopropyl alcohol swab and allowing to air dry prior to placement of the electrodes. sEMG was collected using a 8 channel data acquisition system (PowerLab, ADInstruments, Australia) with Labchart software (ADInstruments, Australia) sampling at 1000 Hz with a 2 MΩ impedance, common mode rejection ratio >110 dB min (50/60 Hz), and noise >5 μV. A bandpass filter (10–500 Hz) was applied prior to digital conversion. Using the same 2 s window as the force analysis, mean root mean square (RMS) of the sEMG was determined using a window width of 50 ms and then a mean value was calculated. Analysis of these values was performed in two separate ways; first, they were normalized to baseline and reported as a percentage and second, the absolute mV Biceps brachii values were divided by absolute mV Triceps brachii to provide a co-contraction ratio.

### Multi-joint experiment

#### Procedures

The warm up for this experiment included low-intensity cycling for 5 minutes, followed by 5 minutes of self-selected dynamic stretching. Participants were positioned on a force plate while holding a lightweight (0.4 kg) aluminium bar across their shoulders. As an extension of the warm up, participants completed 10 submaximal CMJ equal to ~50% of their perceived maximal height, and then one CMJ equal to ~80% of their perceived maximum. Similar to the single joint experiment, participants then completed two CMJs separated by 30 s per condition and two minutes of rest between conditions. The instructions provided to each participant in this experiment are described in Table 1 for each of the investigated conditions. In the Mirror condition a 1.76 by 0.56 m mirror was placed ~2 m away from the centre of the force plate (Fig 1B) whereas in all other conditions a large mono-tone board was placed in front of participants to block out potential vision confounders. In contrast to the more controlled routine of the single-joint experiment in which participants could only see their elbow flexors, in this experiment participants were free to choose what they would look at in the mirror. Other than the single instructional sentence no other guidelines, encouragement, verbal or visual feedback were provided. Finally, after the completion of this experiment, participants answered a questionnaire on their preferred instruction and reported if the neutral-mirror instruction elicited a stronger EF or IF response for both the single and multi-joint experiments (see below).

#### Countermovement jumps

The countermovement jump (CMJ) trials were completed on a commercially available portable force plate (9290AD Quattro Jump, Kistler, Switzerland). Additionally, a single linear position transducer (Ballistic Measurement System, Fitness Technology, Adelaide, Australia) was mounted directly above the participant and utilised to directly measure displacement via a tether attached to the centre of the aluminium pole held by the participant across their shoulders during each CMJ trial. The force plate and a linear position transducer were synchronised and interfaced with a personal computer via an 8 channel data acquisition system (PowerLab, ADInstruments, Australia) with Labchart software (ADInstruments, Australia) sampling at 1000 Hz, allowing direct measurement of force-time characteristics. Ground reaction forces and linear position transducer were analysed using Labchart software and custom macros. Prior to all data collection, the force plate was calibrated using a range of known loads and the linear position transducer was calibrated using a two point calibration process and a known distance. The utilisation of the aluminium bar across the shoulders eliminated arm swing from the movement and thus our outcome measures provide a reflection of only lower body performance capabilities and not the general vertical jumping capacity. Due to the expected large inter-subject variability between genders in maximal jump height, and due to a possible order effect resulting from completing a repeated number jumps, all mean maximal jump values (cm) were normalized to baseline condition, and thus reported and analysed as a percentage.

### Common procedures

#### Questionnaires

Participants answered a two part questionnaire (S1 File) after the completion of the multi-joint experiment. Participants were asked to rank the four listed instructions in accordance with their preference for eliciting their best performance, with 1 being the most preferred and 4 being the least preferred. Participants were then asked to report if the mirror instructions were perceived as more of an IF or EF. This was achieved by having participants mark a vertical line over a 20 cm horizontal line which had EF listed on the left side, and IF on right side. The distance of the drawn vertical line from the midpoint was then measured with a ruler to provide a quantification of how strongly a participant rated the mirror condition as either IF or EF.

#### Statistical analysis

In the single joint experiment data from the two MVICs completed in each of the five conditions were averaged and used for further analysis. To examine if significant differences existed between the first and second contractions in each of the five conditions, a two-way Analysis of Variance (ANOVA) with repeated measures was conducted (conditions [5] x MVICs [2]). A two-way ANOVA with repeated measures was used to compare the mean normalized forces and EMG activity, between the four conditions, and to investigate if a gender effect exists (instructions [4] x gender [2]). An additional two-way ANOVA with repeated measures was used to compare the order of preferences on normalized force production, and to investigate if a gender effect exists (instruction preferences [4] x gender [2]) on normalized force production. If the assumption of Sphericity was violated, the Greenhouse—Geisser correction was employed with an LSD post hoc test if a main effect was identified or paired t-tests with a Boferroni correction if an interaction was identified. In the multi-joint experiment the data from the two CMJs completed in each of the five conditions were averaged and used for further analysis. Using a similar statistical approach as in the single joint experiment, a two-way ANOVA with repeated measures was conducted to investigate if the first jump differed from the second jump within each condition (conditions [5] x jump height [2]). A two-way ANOVA with repeated measures was used to compare the mean normalized jump height, peak vertical concentric force and peak concentric velocity, between the four conditions, and to investigate if a gender effect exists (instructions [4] x gender [2]). An additional two-way ANOVA with repeated measures was used to compare the order of preferences on jump height, and to investigate if a gender effect exists (instruction preferences [4] x gender [2]) on jump height. A Chi-square test of independence was used to examine differences between the number of participants who rated the Mirror condition as either IF or EF. Statistical significance was accepted as *P*< 0.05 for all tests. Furthermore, 95% confidence intervals (CI) of the mean percent differences and Cohen *d* effect sizes (ES) were reported when appropriate. The magnitudes of these ES were classified as trivial (0–0.19), small (0.20–0.49), medium (0.50–0.79) and large (0.80 and greater) using the scale advocated by Cohen [25].

## Results

### Single-joint experiment

**Fig 2:**
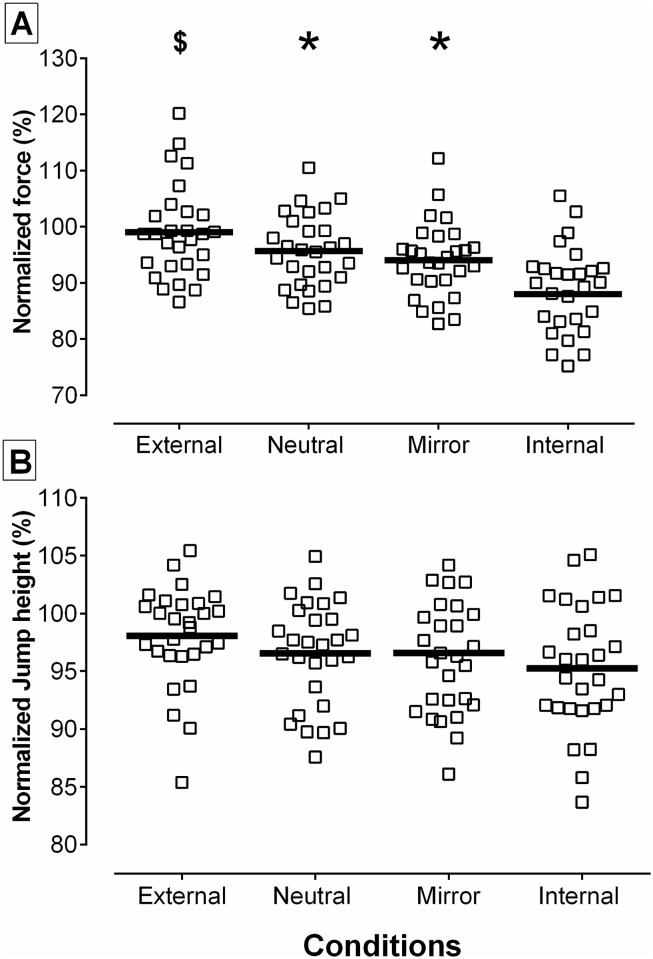
Normalized Performance Measures. (A) MVIC data from single-joint experiment. (B) CMJ data from multi-joint experiment. Note: each square represents data from a single participant and the black horizontal lines represent the group average per condition.

No significant interaction was identified between the first and second MVIC in each of the five conditions (*P* = 0.289). The mean (±SD) absolute force (N) produced in the MVIC in each of the conditions were: EF (268±74 N), IF (240±72 N), Neutral (260±74 N), Mirror (255±72 N) and IF (240±72 N) (S2 File). In this experiment a main effect for instruction type was identified (*P*< 0.001), however no significant interactions were identified between gender and instruction (*P* = 0.741). Specifically, participants produced significantly greater normalized mean force in EF compared to IF (*P*< 0.001; ES = 1.31; CI 95% [6.3, 15.6%]), Neutral (*P* = 0.028; ES = 0.46; CI 95% [0.5, 6.3%]) and Mirror (*P* = 0.017; ES = 0.67; CI 95% [0.9, 8.9%]). When compared to IF, greater normalized force was produced in the Neutral (*P*< 0.001; ES = 0.98; CI 95% [4, 11.2%]), and Mirror conditions (*P*< 0.001; ES = 0.79; CI 95% [2.7, 9.3%]), however, no differences were observed between Neutral and Mirror conditions (*P* = 0.392; ES = 0.14; CI 95% [-2, 5.1%]) (Fig 2A). No significant differences were discerned between the four conditions in normalized sEMG activity of biceps brachii (*P*≥ 0.972), triceps brachii (*P* = 0.588), or co-contraction ratio (absolute mV activity of biceps brachii/ triceps brachii) (*P* = 0.979). The lack of statistical significant sEMG differences were accompanied by small effect sizes (ES≤ 0.12).

**Fig 3:**
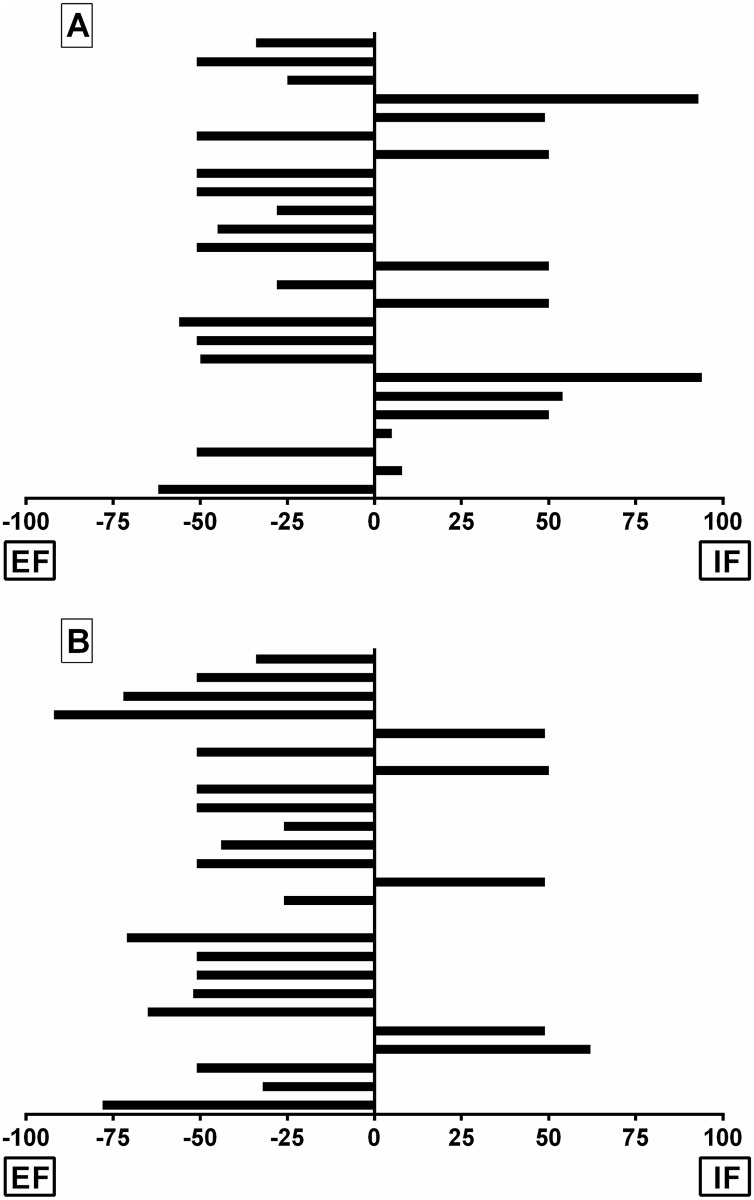
Perception of instructions. Strength of the participants’ perception of the mirror instructions compared to IF and EF in the single-joint (A) and multi-joint (B) experiments.

There were no significant interactions (*P* = 0.445) or main effects (*P* = 0.226) for the participants (n = 25) preferences of instructions on normalized force production (Table 2). That is, the most preferred instruction did not elicit greater force production compared to the least preferred. However, there was a moderate effect (ES = 0.32) for the greatest forces to be associated with the most preferred (EF), compared with other instruction. No differences were seen between the 2^nd^, 3^rd^ and 4^th^ ranked instructions (ES≤ 0.01). No significant differences (X_2_ = 1.01, *P* = 0.297) were observed between the number of participants who rated the Mirror condition as an EF (n = 15), compared to participants who rated it as an IF (n = 10). The strength of the participants’ perception of how the mirror instructions compared to IF and EF is illustrated in Fig 3A.

**Table 2:** Instructional preferences.

	Preference rankings	External	Neutral	Mirror	Internal
Single-joint					
	**1**^**st**^	12	3	5	5
	**2**^**nd**^	6	5	8	6
	**3**^**rd**^	7	5	6	7
	**4**^**th**^	0	12	6	7
Multi-joint					
	**1**^**st**^	15	2	5	3
	**2**^**nd**^	3	9	3	10
	**3**^**rd**^	5	7	7	6
	**4**^**th**^	2	7	10	6

Participants’ preferences of the four instructions in both experiments.

### Multi-joint experiment

A significant interaction was identified between the first and second jump completed across conditions (*P* = 0.042), however the Bonferroni corrected t-test post hoc comparisons were not statistically different within conditions. The mean (±SD) absolute jump heights for all conditions were as follows: EF (38.2±7.4 cm), Neutral (37.6±7.6 cm), Mirror (37.7±7.9 cm) and IF (37.2±7.0 cm) (S2 File). No significant interactions between gender and instructions (*P* = 0.346), or a main effect for instructions were identified (*P* = 0.101). However, despite the lack of statistical differences, the results of this experiment followed a similar pattern to the single-joint experiment in which EF led to greater jump height compared to IF (ES = 0.48; CI 95% [0.36, 4.3%]) and to slightly higher jump heights compared to Neutral (ES = 0.27; CI 95% [-0.41, 3.0%]) and Mirror conditions (ES = 0.26; CI 95% [0.36, 4.3%]) (Fig 2B). No differences were observed between Neutral and Mirror conditions (ES = 0.01; CI 95% [-1.81, 1.92%], but compared to IF, slightly greater jump heights were observed with both Neutral (ES = 0.22; CI 95% [-0.46, 2.90%]) and Mirror (ES = 0.20; CI 95% [-0.57, 3.21%]) conditions. No significant or meaningful differences were identified between the four conditions for normalized peak force (*P* = 0.402), mean force (*P* = 0.670) and mean velocity (*P* = 0.447). The lack of significant differences was accompanied by small effect sizes (ES≤ 0.19). However, peak velocity was statistically significant between conditions (*P* = 0.018) with EF resulting in greater peak velocities compared to IF (*P* = 0.01; ES = 0.19; 95% [0.015–0.108 ms^-2^) and compared to Mirror (*P* = 0.02; ES = 0.13; 95% [0.007–0.092 ms^-2^). The Neutral instructions lead to significantly greater peak velocities compared to Mirror (*P* = 0.014; ES = 0.12; 95% [0.010–0.085 ms^-2^) and IF (*P* = 0.037; ES = 0.17; 95% [0.004–0.116 ms^-2^).

There were no significant interactions (*P* = 0.680) in participant instruction preferences (Table 2), however, a main effects for conditions (*P* = 0.038) was identified. Note that questionnaire data is missing from three participants. The differences between the most and least preferred instructions were not matched for jump height performance. That is, jumping performance did not follow the rank of preferred instructions. The third most preferred instruction elicited greater jump heights compared to all other preferences. Specifically, significantly greater jump heights were found compared to the second most preferred instruction (*P* = 0.029; ES = 0.43; CI 95% [0.5, 4.2%]), the fourth (*P* = 0.028; ES = 0.52; CI 95% [0.3, 4.9%]). A significant difference (X_2_ = 4.83, *P* = 0.027) was observed between the number of participants who rated the Mirror condition as an EF (n = 19), compared to participants who rated it as an IF (n = 5). It should be noted that one participant perceived the Mirror as neither EF or IF. The strength of the participants’ perception of how the mirror instructions compared to IF and EF is illustrated in Fig 3B.

## Discussion

The primary goals of the two experiments were to examine how performance of an isometric single-joint, and a dynamic multi-joint tasks would be affected by performing in front of a mirror; and compare the mirror performance results to the well investigated EF and IF instructions. Elbow flexion forces were greater with EF and the lowest with IF, and a similar trend, albeit not statistically significant, was found with jump heights. Furthermore, in both studies performance in the Mirror conditions were comparable to the Neutral condition. That is, both the Mirror and Neutral conditions were lower than EF but greater than IF. The secondary goals of these experiments were to investigate if participants’ preferences of instructions match their performance; to descriptively analyse if participants perceived the mirror as EF or IF; and examine if a gender effect would be observed. The stated preferences of instructions were not matched with either elbow flexion forces or with jump performance; the majority of participants perceived the mirror to elicit an external focus, although the strength of perception of the mirror differed widely between participants and experiments. Finally, no gender effect was observed in either experiment.

The differences in performance observed in this study are aligned with previous work, in which EF leads to superior performance and IF results in inferior performance [2]. Furthermore, within this study the mirror condition did not seem to result in a reduction or improvement in performance. These results support some [18, 21], but not all [16, 17], studies investigating the effect of mirrors on motor activities. Note that the majority of studies to date that have investigated the influence of a mirror on performance during a motor task have compared it solely to a Neutral condition [11, 13, 15, 18]. In contrast, in the present study, the use of a mirror was also compared to EF and IF conditions which extend our understanding of how mirrors affect performance in a relation to the well-established focus conditions. Further, while previous mirror studies have investigated outcome measures such as balance [16], accuracy [17], movement economy [14] and motor learning [15], to our knowledge no study investigated a maximal force and jumping tasks as in the present study. Investigating such tasks is important as both trained and untrained participants commonly perform resistance training exercises in front of mirrors in gym environments. While the presence of a mirror may be of value in movement tasks requiring accuracy, such as squat depth assessment [11], our study indicates that the mirror does not provide meaningful benefits in activities requiring maximal force and in jumping performance. Interestingly, however, sEMG of both the agonist and antagonist muscle groups did not differ between any conditions in the single-joint experiment which is in contrast to previous attentional focus research on the elbow flexors [6, 26]. These contrasting findings may be in part due to differences in signal normalization techniques, as well as the difference in implemented contraction types. Whereas in the present study isometric contractions were used, dynamic contractions were used in the two previous studies.

The results from both experiments indicate a lack of relationship between the preference of instruction and performance outcomes. That is, irrespective of how participants ranked their preferences for the four instructions in both studies, force and jump height remained unaffected. This supports the previous work of Wulf et al. [27], in which a balance task was completed with fewer errors with EF irrespective if participants preferred IF or EF. Other authors have reported that participants’ preferences of IF and EF influence their performance to some extent in tasks requiring accuracy, such as dart throwing, billiards and basketball throws [8, 9, 28]. However, within the studies investigating the relationship between attentional instructions and participants preferences, the benefits of EF persist despite situations where use of a non-preferred focus condition was imposed. That is, performance of participants who preferred EF but were asked to use their non-preferred IF suffered to a greater extent compared to those who preferred IF but were asked to use their non-preferred EF [8, 9, 28]. Thus, while preferences of focus conditions can account for some of the effects on performance, it seems as if performance is affected to a greater extent by the type of focus instruction adopted.

Similar to other studies [8, 27], participants in both experiments generally ranked EF as their most preferred focus conditions (Table 2). Whereas a considerable range of perceptions were reported regarding the degree to which the Mirror condition elicited IF and EF in the single-joint experiments (Fig 3A), most participants in the multi-joint experiment reported that the Mirror was perceived more as an EF rather than IF (Fig 3B). This observation is interesting as mirrors can be expected to either; 1) elicit a self-conscious response and thereby lead to IF, or 2) to shift participants focus away from themselves as they observe the mirror and thereby elicit an EF. We speculate that the variation of individual response may be reflective of training and life experiences. Future investigations should seek cohorts of participants that could be initially classified on sporting skill level or experience in an environment to continue to refine our understanding on the use of mirrors as an instruction focus tool.

The *constrained action hypothesis* proposed by Wulf et al. [29] provides an explanation of our observed differences in performance between EF and IF in both experiments. In this regard, it is hypothesized that EF allows participants to self-organize in an automatic manner and perform the task unconstrained by conscious control. Conversely, IF disrupts the automaticity of performance, making participants conscious of their movements. Although not overtly evident in the single joint experiment, performing an MVIC required participants to stabilise and synchronise their shoulder and trunk muscles as they performed the contraction. The requirement to synchronise and coordinate numerous body parts and muscle groups to elicit optimal performance is more evident in the multi-joint experiment. Thus, we speculate that IF leads participants to focus on a single component of a complex movement task, which reduces the contribution of other body parts and muscle groups, thereby hindering performance. In contrast, EF allows participants to organize the relevant contributors around the motor task without neglecting any one of the contributors in a more natural organisation of the motor pathway.

Our observation that the mirror condition was more neutral in the performance effect cannot be neatly explained by the *constrained action hypothesis*. However, given the inter-individual perception of the mirror condition as either IF and EF, we speculate that the *constrained action hypothesis* can account for both the negative and positive effects as a function of the mirrors perception as IF or EF. In cases in which the mirror elicit a negative effect then the use of a mirror is inducing a partial IF response, and in contrast, when participants focus on what they observe in the mirror as external to the self, a partial EF response results. Future studies should utilize specific IF and EF instructions as participants observe their movement within a mirror to enhance our understanding of the *constrained action hypothesis*.

It would be remiss if we did not consider the impact of our imposed experiment design constraints on the observed outcomes. An important consideration within this study was our decision to not counterbalance the order of the two experiments due to logistical constrains which could have led to an order effect or bias participants’ expectations. While we did seek to compare the magnitude of response between conditions, the smaller effect sizes observed in the multi-joint experiment could be related the order in which the experiments were conducted. There is also the possibility that participants did not receive adequate familiarization with the motor tasks. Particularly, there were some inter-individual differences related to participants experience with the jumping task. These experience differences between participants could partially account for the smaller effects observed in the second experiment. Finally, the preference questionnaires for both experiments were conducted only after the completion of the multi-joint experiment. Thus, this elapsed time between the completion of the single-joint experiment and the questionnaires completion could have somewhat skewed the results. However, not doing so would have compromised the efficacy of the second experiment.

## Conclusion

We have reported that EF leads to superior performance in both a single and multi-joint tasks compared to all conditions, and that IF leads to inferior performance in such tasks. The Mirror condition led to inferior performance compared to EF, superior performance compared to IF and was comparable to the Neutral conditions. A lack of relationship between participants’ preferences of instruction type to performance outcomes was observed, as well as a wide range of responses pertaining to how the Mirror condition was perceived in relation to IF and EF. Finally, the effects were similar between males and females. We emphasised internal validity in the single-joint experiment and external and ecological validity in the multi-joint experiment. Since the results followed a similar pattern in both experiments, we consider these findings to be robust. These results are of practical relevance given the popularity of training in front of mirrors in studios and gyms, and also expand our understanding of how focus conditions influence performance.

## Supporting Information

S1 File Questionnaire. (PDF)

S2 File Absolute values for both experiments. (XLSX)
