# Supplementary material for: The Effects of Either a Mirror, Internal or External Focus Instructions on Single and Multi-Joint Tasks
Source: PLoS One. 2016 Nov 29;11(11):e0166799. doi: 10.1371/journal.pone.0166799 (PMC5127520; doi:10.1371/journal.pone.0166799)
Supplement: S1 File — (PDF) [file pone.0166799.s001.pdf]

Name: _______________

Biceps

Rank from 1 to 4 with 1 being the most preferred instructions and 4 being the least preferred instructions.

	**Internal**: When you attempt to produce as much force as you as you possibly can, I want you to focus on contracting your arm muscles as hard and as fast as you can.
	**External**: When you attempt to produce as much force as you possibly can, I want you to focus on pulling the strap as hard and as fast as you can.
	**Control**: attempt to produce as much force as you as you possibly can.
	**Mirror:** attempt to produce as much force as you as you possibly can while looking at yourself in the mirror.

Where did you perceive the mirror instructions to be relative to "Internal" or "External"

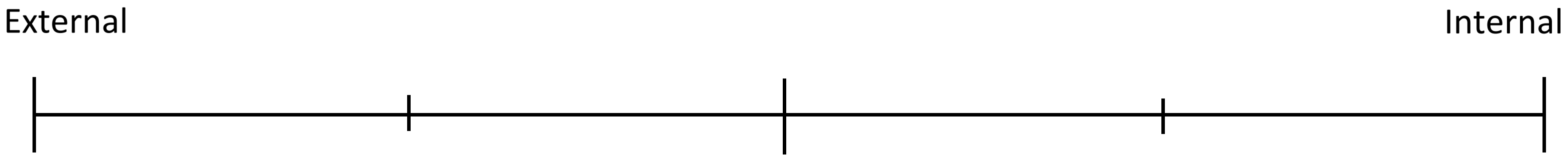

Jumps

Rank from 1 to 4 with 1 being the most preferred instructions and 4 being the least preferred instructions.

	**Internal:** When you attempt to jump as high as you possibly can, I want you to focus on extending your legs as hard and as fast as you can.
	**External:** When you attempt to jump as high as you possibly can, I want you to focus on pushing off the ground as hard and as fast as you possibly can.
	**Control:** attempt to jump as high as you possibly can.
	**Mirror:** attempt to jump as high as you possibly can while looking at yourself in the mirror.

Where did you perceive the mirror instructions to be relative to "Internal" or "External"

External Internal
